# Spontaneous Thrombosis of a Hepatic Pseudoaneurysm Following Blunt Liver Injury

**DOI:** 10.7759/cureus.39453

**Published:** 2023-05-24

**Authors:** Hiroshi Fukumasa, Shingo Niimi, Masashi Kobayashi, Toshihito Uehara, Kohji Okamoto

**Affiliations:** 1 Pediatrics, Kitakyushu City Yahata Hospital, Kitakyushu, JPN; 2 Surgery, Kitakyushu City Yahata Hospital, Kitakyushu, JPN

**Keywords:** spontaneous thrombosis, conservative management, pseudo aneurysm, trauma pediatric, blunt liver trauma

## Abstract

Hepatic pseudoaneurysm (HPA) is a rare complication of liver injury in children. Prophylactic embolization is preferable to prevent life-threatening hemorrhage due to pseudoaneurysm rupture. We present the case of a four-year-old boy who sustained a grade III liver injury from blunt abdominal trauma. He was conservatively managed since he was hemodynamically stable. Follow-up contrast-enhanced computed tomography (CECT) performed 10 days following the injury revealed an HPA measuring 4 mm × 4 mm × 3 mm. Herein, we chose conservative treatment for HPA as the patient was asymptomatic and hemodynamically stable. Conservative treatment was successful, and HPA spontaneously resolved 23 days following the injury without radiologic or surgical intervention. Although there are studies reporting asymptomatic HPAs that have spontaneously resolved, the natural history of HPAs remains unknown. Conservative treatment may be an option for asymptomatic HPA; however, to identify factors contributing to spontaneous thrombosis, further evaluation is needed.

## Introduction

A liver injury is one of the most common pediatric abdominal injuries due to blunt abdominal trauma. Post-traumatic hepatic pseudoaneurysms (HPAs) are rare complications in these children [1]. However, HPA should not be ignored owing to the life-threatening risk of bleeding due to rupture [1-4]. Therefore, prophylactic embolization has been recommended in some studies [5-7]. Nevertheless, there have been reports of HPAs spontaneously resolving with conservative treatment without intervention [8-10]. Here, we present the case of a four-year-old boy with HPA exacerbated after a grade III liver injury according to the criteria of the American Association for the Surgery of Trauma (AAST) liver injury scale due to abdominal blunt trauma, which was resolved by spontaneous thrombosis with elected conservative treatment.

## Case presentation

A four-year-old boy presented to our clinic with blunt abdominal trauma caused by his mother’s elbow striking his abdomen when he was stuck beneath her as she fell backward. On the initial examination, he presented with mild upper abdominal tenderness without nausea or vomiting. Cardiac and respiratory examinations were normal. There were no abnormal findings, such as subcutaneous hematomas of the skin. The following were his vital signs on admission: Glasgow coma scale, 15 (E4V5M6); heart rate, 129 beats/min; blood pressure, 81/55 mmHg; respiratory rate, 36/min; oxygen saturation in room air, 99%; and body temperature, 37.1 °C. Hematology test results showed that the hemoglobin level and the platelet count were within the normal range. His aspartate aminotransferase, alanine transferase, lactate dehydrogenase, and D-dimer levels were elevated (Table 1).

**Table 1:** Laboratory analyses PT-INR: prothrombin time-international normalized ratio, APTT: activated partial thromboplastin

Parameter	Value	Normal range
White cell count (cells/μL)	15,000	3300–8600
Hemoglobin (g/dL)	12.3	13.7–16.8
Platelet count (cells/μL)	278,000	158,000–348,000
Total bilirubin (mg/dL)	0.3	0.4–1.5
Aspartate aminotransferase (U/L)	478	13–30
Alanine transferase (U/L)	220	10–42
Lactate dehydrogenase (U/L)	607	124–222
Amylase (U/L)	92	44–132
PT-INR	1.00	0.9–1.3
APTT (seconds)	21.9	24–32
Fibrinogen (mg/dL)	261	200–400
D-dimer (μg/ml)	5.68	≦1.0

**Figure 1:**
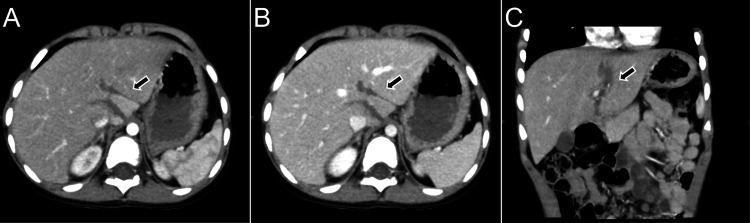
Initial contrast-enhanced computed tomography of the abdomen The initial CECT performed on the day of injury showing grade III laceration of segments V and I of the liver, representing deep hepatic injury with no extravasation (black arrow). (A) Axial view of CECT in the late arterial phase, (B) axial view of CECT in the portal phase, and (C) coronal view of CECT in the late arterial phase.

A focused assessment with sonography for trauma revealed a small volume of fluid collection in the rectovesical pouch. Abdominal contrast-enhanced computed tomography (CECT) revealed a grade III liver injury according to the criteria of the AAST liver injury scale with a laceration of segments IV and I of the liver without active extravasation (Figure 1). As his blood pressure and hemoglobin levels were stable and his abdominal pain did not worsen, he was conservatively managed.

**Figure 2:**
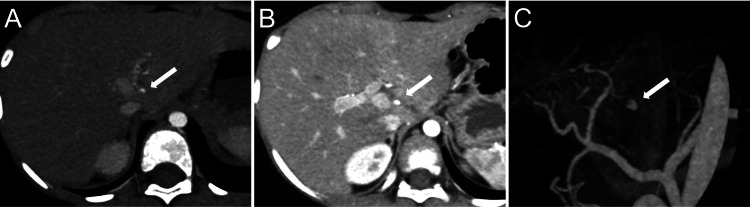
Follow-up contrast-enhanced computed tomography performed 10 days following the injury (A) Axial view of CECT in the early arterial phase, (B) axial view of CECT in the portal phase, and (C) three-dimensional CT angiography, showing a hepatic pseudoaneurysm (HPA) measuring 4 mm × 4 mm × 3 mm (white arrow).

Doppler ultrasound (DUS) performed two and five days following the injury showed a hypoechoic area within segments IV and I, with no findings indicative of intrahepatic biloma or HPAs. Follow-up CECT performed 10 days following the injury revealed a stained area (4 mm × 4 mm × 3 mm) confined to the hepatic hilar region early in the arterial phase and enhanced staining late in this phase (Figure 2).

Therefore, a diagnosis of HPA was made. We initially considered prophylactic angiographic embolization for treating the HPA; however, we opted for conservative treatment for the following reasons: asymptomatic HPA with no abnormal vital signs and further pain; the relatively small size of the HPA, which is located within the Glisson’s sheath and surrounded by the hepatic parenchyma; and no extravascular leakage or side effects, including hepatic necrosis and the invasiveness of the embolization. While maintaining readiness for urgent therapeutic intervention, if necessary, he was continually conservatively managed and received inpatient care. A periodic DUS with weekly follow-up revealed no signs of HPA rupture.

**Figure 3:**
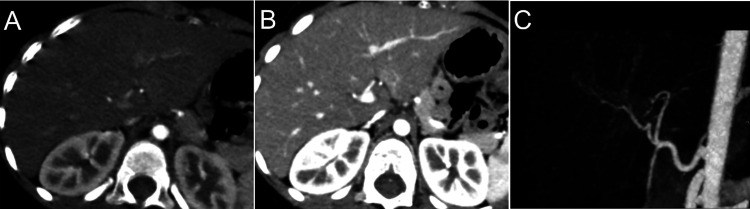
Second follow-up contrast-enhanced computed tomography performed 23 days following the injury (A) Axial view of CECT in the early arterial phase, (B) axial view of CECT in the late arterial phase, and (D) three-dimensional CT angiography, showing hepatic pseudoaneurysm disappearance.

A repeat CECT performed 23 days following the injury revealed no HPA. The liver laceration was almost completely resolved to a hair-like, fine, linear, low-density area on CECT (Figure 3). We assumed that the HPA was resolved by spontaneous thrombosis. On day 25 of hospitalization, the patient was discharged. A year later, a follow-up at an outpatient clinic revealed that he was asymptomatic.

## Discussion

Nonoperative management of blunt liver injury in children has already been established, and guidelines have been published [11]. However, the need for screening imaging studies for complications is controversial. The ASPA trauma committee [11] and other reports [12,13] do not recommend imaging follow-up for asymptomatic patients with liver injury but only for those with symptoms. In particular, previous studies have reported that the incidence of post-traumatic HPA was 3-4% in adults [2,3]. It is even rarer in children, with a reported incidence of 1.7% [1]. These very low incidences of HPA may be a factor in which routine follow-up imaging for asymptomatic children with blunt liver injury is not warranted.

However, Safavi et al. later found that routine surveillance imaging studies of patients with liver injury may be warranted because of the potential risk of hemorrhage from HPA rupture [1]. According to Østerballe et al., follow-up CTs for liver injury with conservative treatment would be appropriate to avoid HPA rupture [3]. Moreover, Wanger et al. reported missing asymptomatic HPAs because follow-up imaging studies after the initial evaluation of trauma in adult cases were not conducted. They proposed routine surveillance five to seven days after injury, particularly for high-grade liver injuries [2]. Durkin et al. reported that at follow-up screening 5-10 days after injury, HPA occurred in 14/57 liver injury cases in children (25%) regardless of the grade of injury or the presence or absence of any symptoms, including worsening abdominal pain, hemodynamic instability, and decreased hemoglobin levels [10]. Although there are no uniform criteria for follow-up imaging studies for patients with liver injury at our hospital, all patients with liver injury are followed up using US and CECT during hospitalization. Herein, a follow-up CECT performed 10 days after the injury revealed HPA; however, the patient was asymptomatic. Therefore, it may be appropriate to perform routine surveillance imaging studies for traumatic liver injury 5-10 days after injury, and as per Durkin et al. [10], routine imaging screening may reveal a higher frequency of HPA after trauma than previously reported.

To prevent life-threatening hemorrhage due to HPA rupture, prophylactic embolization is preferable [5-7]. Conversely, some studies have reported the resolution of asymptomatic HPA with spontaneous thrombosis. Soudack et al. followed up on three HPAs in a 4-year-old girl using DUS and CECT, revealing partial thrombus formation at 2 weeks and complete occlusion 1.5 months after the injury [9]. Shava et al. followed up HPA in a 7-year-old girl using DUS, revealing partial thrombosis at 3 weeks and complete disappearance at 4 weeks after the injury [8]. Durkin et al. reported that of seven children who developed asymptomatic HPAs following liver injury, six had spontaneous thrombosis and resolution within a median of 13 (10-18) days following the injury using unenhanced US, CECT, or contrast-enhanced US [10]. Although Wagner et al. reported four adult cases of spontaneous thrombosis of asymptomatic HPA using CECT, they stated that spontaneous thrombosis of pseudoaneurysms has only been anecdotally reported in the literature [2]. To the best of our knowledge, the true rate of spontaneous thrombosis without intervention is unknown, and there have been no known factors that influence asymptomatic HPA rupture or spontaneous thrombosis. The criteria for HPA embolization used at King’s College Hospital in London, the United Kingdom, may be helpful for determining what makes conservative treatment for HPA successful [10]. Their criteria include an HPA >10 mm or bile leakage. In their series, thrombosis or resolution was documented in six of seven cases (86%) with asymptomatic HPA. One child (14%) was asymptomatic, although he was embolized after meeting the institutional criteria. Therefore, as a parameter for conservatively treating asymptomatic HPAs, it may be necessary to confirm that a pseudoaneurysm size is not >10 mm or that there is a trend of reduction in the pseudoaneurysm size at least two to three weeks after an injury during conservative treatment with close monitoring as inpatients. However, the actual incidence is unknown, and their natural history remains unclear. Therefore, based on our experience in this case, we do not recommend conservative treatment for all asymptomatic HPAs. If signs of HPA enlargement, no reduction in HPA size with spontaneous thrombus formation, or any symptoms, including worsening abdominal pain, abnormal vital signs, or decreased hemoglobin levels, are observed during follow-up, embolization should be considered.

## Conclusions

Due to thrombosis, post-traumatic asymptomatic HPAs may resolve spontaneously. Further research is required to determine which cases can be expected to have spontaneous thrombosis based on the liver injury grade and the pseudoaneurysm size under the assumption that patients will be safely managed and how long the conservative treatment can be tolerated.
